# Bioactive Oxylipins Profile in Marine Microalgae

**DOI:** 10.3390/md21030136

**Published:** 2023-02-22

**Authors:** Amandyne Linares-Maurizi, Guillaume Reversat, Rana Awad, Valérie Bultel-Poncé, Camille Oger, Jean-Marie Galano, Laurence Balas, Anaelle Durbec, Justine Bertrand-Michel, Thierry Durand, Rémi Pradelles, Claire Vigor

**Affiliations:** 1 Institut des Biomolécules Max Mousseron, IBMM, Université de Montpellier, CNRS, ENSCM, 34093 Montpellier, France; 2 Microphyt, 713 Route de Mudaison, 34670 Baillargues, France; 3 MetaToul, MetaboHUB, Inserm/UPS UMR 1048, I2MC, Institut des Maladies Métaboliques et Cardiovasculaires, 31077 Toulouse, France

**Keywords:** microalgae, oxidative stress, oxylipins, LC-MS/MS, lipidomic

## Abstract

Microalgae are photosynthetic microscopic organisms that serve as the primary food source in aquatic environments. Microalgae can synthesize a wide variety of molecules, such as polyunsaturated fatty acids (PUFAs) of the omega-3 and omega-6 series. Oxidative degradation of PUFA due to radical and/or enzymatic conversion leads to the formation of oxylipins, which are compounds known for their bioactive properties. In the present study, we aim to profile oxylipins from five microalgae species grown in 10-L photo-bioreactors under optimal conditions. During their exponential phase, microalgae were harvested, extracted and analyzed by LC-MS/MS to determine the qualitative and quantitative profile of oxylipins for each species. The five different selected microalgae revealed a high diversity of metabolites, up to 33 non-enzymatic and 24 enzymatic oxylipins present in different concentrations. Taken together, these findings highlight an interesting role of marine microalgae as a source of bioactive lipids mediators, which we hypothesize have an important function in preventive health measures such as amelioration of inflammation. The rich mixture of oxylipins may display advantages to biological organisms, especially by providing for human health benefits including antioxidant, anti-inflammatory, neuroprotective or immunomodulator activities. Some oxylipins are also well known for their cardiovascular properties.

## 1. Introduction

Microalgae are the first photosynthetic eukaryotic organisms existing on earth. Microalgae appeared approximately 1.5 billion years ago via the endosymbiosis phenomenon, or the engulfment of bacteria by prokaryotic organisms [1]. Microalgae are autotrophic unicellular organisms that develop in moist environments. Despite making up only 2% of the planet’s biomass, they contribute 50% of the world’s atmospheric oxygen [2]. Being at the base of the food chain and taking part in both carbon cycle and biochemical cycles, they play a significant role in the marine ecosystem [3].

Microalgae have been recently implicated in several biotechnological applications especially in the production of high-value compounds, such as sterols, vitamins, proteins, pigments and lipids [4]. This is due to high amounts of lipid production in microalgae, which is up to ten times higher than that in terrestrial plants [5]. Among the lipids formed, some of them are essential components of healthy diet; these are the bioactive marine polar lipids bearing unsaturated fatty acids (UFAs) including monounsaturated fatty acids (MUFAs) and polyunsaturated fatty acids (PUFAs). They are believed to be a great source of energy, providing essential fatty acids for the body. Because they are more stable and more bio-disponible than triglycerides, they are credited with many health benefits. These lipids are thought to reduce inflammation, support brain health and heart functions, and even help protect against certain diseases [6]. Their bioactivity would be made possible in part by their transfer from the cell membrane to the intracellular compartment where they can be metabolized by enzymes (COX, LOX) to produce oxylipins. For now, more specifically, let us consider the PUFAs present in microalgae [7].

We aim to investigate the production of omega-3 and -6 polyunsaturated fatty acids (PUFAs) in microalgae, including linoleic acid (LA), arachidonic acid (ARA), docosapentaenoic acid (DPA), alpha-linolenic acid (ALA), eicosapentaenoic acid (EPA) and docosahexaenoic acid (DHA) [8]. Algae are known as the only photosynthetic organisms capable of synthesizing the three omega-3 PUFAs (ALA, EPA and DHA) and are the main dietary source of omega-3 in oily fish [9].

Whereas these PUFAs have been reported to display several biological activities, their oxidized metabolites are biologically active, and evidence supports their contribution to beneficial properties of PUFAs [10,11,12].

PUFAs are highly reactive species susceptible to oxidation due to the presence of bis-allylic systems with particularly labile protons in their carbon skeleton [13]. Oxidative stress (OS), the main biological perturbance responsible for the peroxidation of PUFAs, leads to the formation of pro-oxidant species that interact with PUFAs to produce oxidized derivatives obtained either enzymatically or non-enzymatically, and are known as oxylipins [14].

Oxylipins produced by non-enzymatic oxidation PUFA are also known as isoprostanoids or NEO-PUFAs (Non-Enzymatic Oxidized PUFAs) [15] and are at least as diverse as their enzymatic counterparts. Oxylipins are distinguished by their oxidation mechanism, which is a free radical-based process that is not initiated by an enzymatic workflow-although enzymes could be the origin of the reactive oxygenated species (ROS) responsible for their formation [16]. As a result, several PUFA metabolites are produced, with each able to produce a variety of oxidized derivatives with different structures. For example, it is estimated that ALA can lead to the formation of 32 oxidized metabolites called F_1t_-Phytoprostanes, ARA to 64 metabolites (F_2t_-Isoprostanes), EPA to 96 metabolites, (F_3t_-Isoprostanes) and DHA to 128 metabolites (neuroprostanes) [16], while the enzymatic version would produce only one F-type; a cyclopentane ring with two alcohol functions, per PUFAs. Furthermore, unique structures can be non-enzymatically generated such as in furanoids, which are characterized by a tetrahydrofuran ring with two side chains, increasing the metabolic diversity of non-enzymatic oxidation products of PUFAs [17].

Enzyme derivatives have unique and specific isomers since they are produced by enzymatic catalysis. Oxylipin biosynthesis is initiated by the action of dioxygenases from cyclooxygenase (COX1 and COX2) or lipoxygenase (LOXs) type [18]. The produced hydroperoxides and/or endoperoxides are produced, which can then be converted by the cytochrome (CYP) P450 family into prostaglandins, lipoxins, resolvins, protectins, and linotrines [19]. The natural synthesis of oxylipins involves some enzymatic pools known as specialized proresolving mediators (SPMs), which have a crucial role in the resolution of inflammatory processes [20]. Some derivatives of LA and ARA have been shown to regulate endogenous inflammation levels [21,22]. The lipoxygenase-catalyzed derivatives of ALA, such as EPA (resolvins E) and DHA (resolvins D, protectins), have been reported to display anti-inflammatory properties [23] in nano and picomolar levels [24,25]. Among the other biological activities of DHA-derived molecules, these molecules can promote tissue regeneration [26] or alleviate post-operative pain [27].

Similar to enzymatic oxylipins, non-enzymatic oxylipins are involved in a variety of biological functions such as inflammatory and immune responses associated with pathologies [16,17,28]. Some ARA metabolites (F_2t_-IsoPs) have been shown to exert a vasoconstrictor effect [29,30] and anti-inflammatory effects [31,32] in numerous organs. Neuroprotection [33] and the allergic responses [34] have been associated with the phytoprostanes, which are ALA derivatives. Additional studies have demonstrated cardiovascular and neuroprotective effects of F_3t_-Isoprostanes and neuroprostanes DHA derivatives [35,36,37,38].

**Figure 1:**
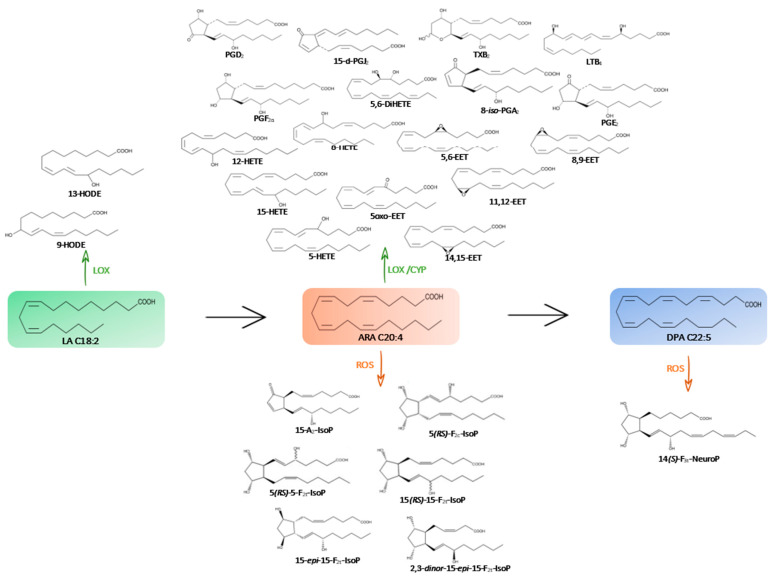
Metabolic pathway of omega-6 PUFAs (→) and their oxygenated derivatives (enzymatic pathway in green with CYP, LOX and non-enzymatic pathway in orange with ROS) in the microalgae species studied.

**Figure 2:**
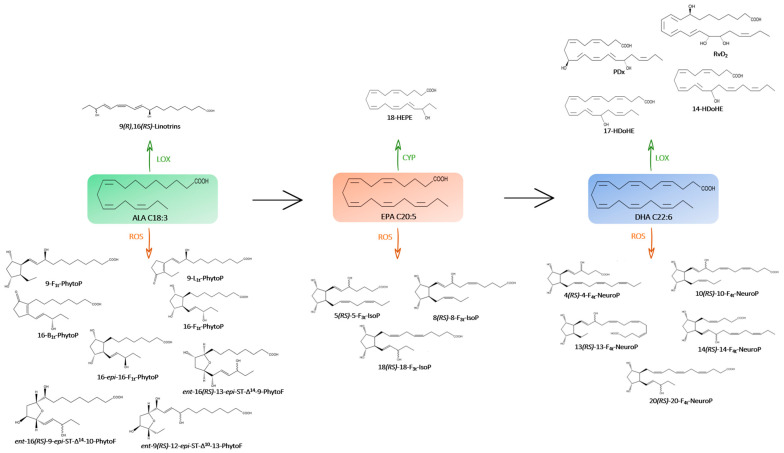
Metabolic pathway of omega-3 PUFAs (→) and their oxygenated derivatives (enzymatic pathway in green with CYP, LOX and non-enzymatic pathway in orange with ROS) in the microalgae species studied.

Figure 1 and Figure 2 show the main structures found in the microalgae species studied.

Several studies have reported the presence of non-enzymatic [39,40] and enzymatic [41,42] oxylipins in macroalgae [43]. Recently, non-enzymatic oxylipins in microalgae have also been quantified [44]. Our previous work [45] has established a broad profile of non-enzymatic oxylipins in five different species of marine microalgae.

The current study aimed to profile a wider range of secondary oxidation products, including both enzymatic and non-enzymatic oxylipins, within the five microalgae (Mi124, Mi133, Mi134, Mi136, Mi168) that were grown under optimal conditions. This work is the result of a larger scale production (at 10 L scale), which is greater than typical studies on plant material, but will ultimately assist in achieving production under industrial conditions.

## 2. Results

In this study, we highlighted the oxylipins derived from omega-6 lipids, starting with LA, ARA and DPA derivatives. For each of them, we distinguished between enzymatic and non-enzymatic oxylipins. The same process was used for the omega-3 derivatives ALA, EPA and finally DHA. The results of biological triplicates were expressed in ng/mg of dry-weight biomass (DWB) and the numerical data are summarized in Table S1 and S2. The matrix effect (ME) and the extraction recovery (ER), which can alternatively be expressed as the process efficiency (PE), were assessed for non-enzymatic oxylipins because the solid phase or matrix of the extraction cartridge can alter the quantitative value of the metabolites.

Results are shown in supplementary data (Tables S3–S7). A compound loss was observed in the ER during solid phase extraction (SPE), with an average value ranging from 41.40% for Mi134 (Supplementary Table S5) to 77.71% for Mi136 (Supplementary Table S6). Isoprostanoids are retained by the matrix in amounts ranging from 46.49% with Mi136 (Supplementary Table S6) to 82.45% with Mi168 (Supplementary Table S7). The matrix and the SPE effects contribute to high overall process efficiency, with losses ranging from 28.28% for Mi134 (Supplementary Table S5) and 57.02% for Mi133 (Supplementary Table S4). Thus, Mi133 seems to be the most affected species by the extraction process, leading to the greatest underestimation of the NEO-PUFAs content.

### 2.1. Omega-6 Oxidative Derivatives in Microalgae

#### 2.1.1. Derivatives of LA (C18:2 *n*-6)

Enzymatic oxylipins can be derived from linoleic acid (LA), unlike the non-enzymatic ones, due to the requirement of at least three double bonds for the formation of a cyclic structure. 

**Figure 3:**
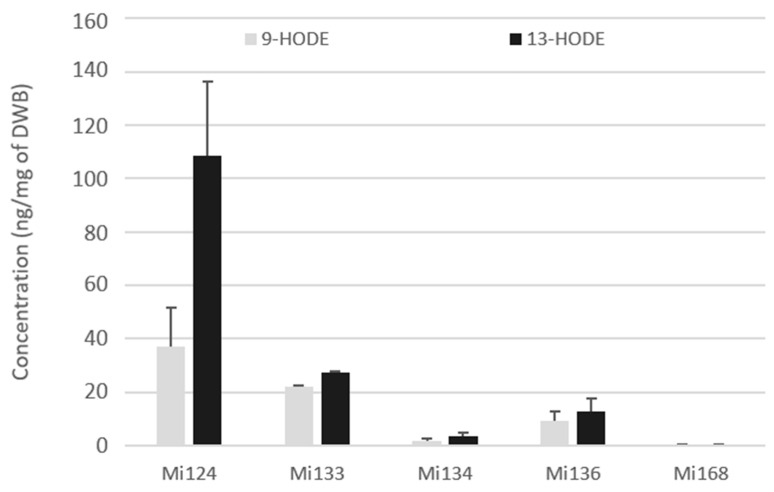
Enzymatic oxygenated metabolites of LA profile in Mi124, Mi133, Mi134, Mi136 and Mi168 expressed in ng/mg (*n* = 3).

The 9- and 13-hydroxy-octadecadienoic acids (HODEs) generated by LOX enzyme (5- and 15-LOX respectively) were quantified in the fresh biomass of five microalgae (Figure 3). 

Both metabolites were present in the five microalgae but in different concentrations (Figure 3). For each microalgae examined, a large amount of 13-HODEs was consistently generated. Mi124 contains HODEs in larger concentration than in Mi133, which are higher than in Mi136. HODEs are present in Mi124 at higher concentration (up to 108.4 ng/mg DWB for 13-HODE and up to 37.1 ng/mg DWB for 9-HODE) than in Mi133 (up to 27.3 ng/mg DWB for 13-HODE and up to 22.0 ng/mg DWB for 9-HODE). Mi136 has a lower concentration than Mi133 (12.8 ng/mg DWB for 13-HODE and 9.2 ng/mg DWB for 9-HODE). The concentration of HODEs in the other two microalgae (Mi134 and Mi136) was less than 3.5 ng/mg of DWB.

In the case of Mi124, 13-HODE concentration was up to three times higher than that of 9-HODE concentration.

#### 2.1.2. Derivatives of ARA (C20:4 *n*-6)

- Enzymatic oxylipins profile

Arachidonic acid (ARA) serves as a precursor of various bioactive compounds using different pathways.

The lipoxygenase (LOX) pathway generates lipoxins (LXs), leukotrienes (LTs), and Hydroxy-Eicosatetraenoic acids (H-ETEs). Epoxyeicosatrienoic acids (EETs) are additionally produced through the cytochrome P450 (CYP) monooxygenase pathway. Moreover, cyclooxygenase (COX) generates prostaglandins (PGs) A, D, E, F and J series as well as thromboxanes (TXBs).

**Figure 4:**
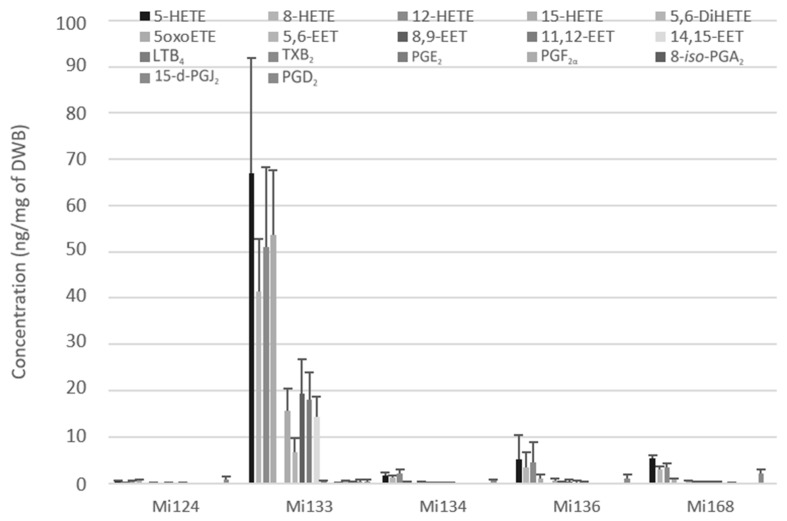
Enzymatic oxygenated metabolites of ARA profile in Mi124, Mi133, Mi134, Mi136 and Mi168 expressed in ng/mg (*n* = 3).

Mi133 revealed the most diversified and abundant profile for all enzymatic ARA-derivatives (288.4 ng/mg) (Figure 4). Among the five microalgae, Mi133 is the species one to produce prostaglandins from the COX pathway such as 8-*iso*-PGA_2_, PGD_2_, PGE_2_, PGF_2α_ except for 15-d-PGJ_2,_ which is present in all five species. The thromboxane TXB_2_ was only detected in Mi168 with less than 0.1 ng/mg (74.9 pg/mg).

Results showed that the microalgae studied contained five H-ETEs and four EETs with concentrations of 2.1 ng/mg for Mi124 DWB, 233.3 ng/mg for Mi133, 6.0 ng/mg for Mi134, 15.4 ng/mg of Mi136 and 14.3 ng/mg for Mi168 (Figure 4).

Notably, for the five species, H-ETEs predominated and were greater than EETs by a factor of 10 (up to 3 for Mi133 and 30 for Mi124) (Figure 4).

Based on this analysis, Mi133 was the most concentrated in ARA derivatives, while TXB_2_ was only found in Mi168.

- Non-enzymatic oxylipins profile

**Figure 5:**
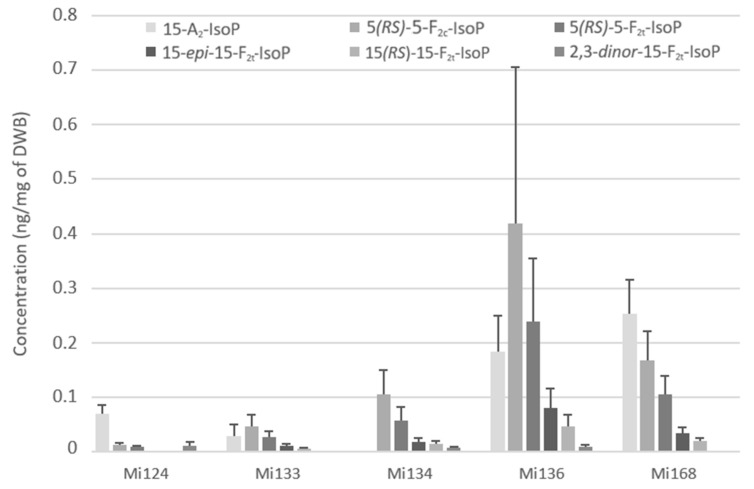
Non-enzymatic oxygenated metabolites of ARA profile in Mi124, Mi133, Mi134, Mi136 and Mi168 expressed in ng/mg (*n* = 3).

ARA are also attacked by the reactive oxygen species (ROS) to produce F_2_ and A_2_- types of isoprostanes. We succeeded in the quantification of the 15-A_2_-IsoP, 5-F_2c_-IsoP, the two diastereoisomers of 5-F_2t_-IsoP, 15-*epi*-15-F_2t_-IsoP, 15-F_2t_-IsoP as well as the 2,3-*dinor*-15-F_2t_-IsoP, an intermediate compound which is produced by ß-oxidation of 15-F_2t_-IsoP. The 2,3-*dinor*-15-F_2t_-IsoP was detected in the lowest concentration (>0.1 ng/mg) compared to other F_2_- and A_2_- types isoprostanes (Figure 5).

Mi136 showed the highest concentration with a total value of 0.9 ng/mg followed by Mi168 which had a total of 0.6 ng/mg of DWB (Figure 5). The other concentrations remained around 0.2 ng/mg for Mi134 and Mi136 and more or less than 0.1 ng/mg for Mi133 (114.6 pg/mg) and Mi124 (99.4 pg/mg). The most abundant IsoP in microalgae, except in Mi124 and Mi168, was 15-F_2c_-IsoP. This compound reached a value of 0.4 ng/mg for Mi136, 0.1 ng/mg for Mi134 and less than 0.1 ng/mg for Mi133 (45.3 pg/mg). The isoprostane of series A is the most prevalent IsoP for Mi124 and Mi168 (Figure 5).

#### 2.1.3. Derivatives of Omega-6 DPA (C22:5 *n*-6)

Two of the non-enzymatic docosapentanoic acid derivatives were investigated and analyzed in marine microalgae: 4-F_3t_-NeuroP and 14*(S)*-F_3t_-NeuroP. Only the presence of 4-F_3t_-NeuroP was detected in the five species, with 0.2 ng/mg in Mi168 and less than 0.1 ng/mg in Mi124, Mi133, Mi134 and Mi136 (44.7 pg, 8.9 pg, 9.9 pg and 18.0 pg/mg respectively) (Supplementary Table S1).

### 2.2. Omega-3 Oxidative Derivatives in Microalgae

#### 2.2.1. Derivatives of ALA

- Enzymatic Profile

Alpha-linolenic acid (ALA), the precursor of eicosapentaenoic acid (EPA) and docosahexaenoic acid (DHA) in microalgae, is an essential omega-3 PUFA that the human body is unable to produce. This omega-3 is a peroxidation target for 15-lipoxygenase and produces linotrins [23].

The 9*(R)*,16*(R,S)*-linotrin diastereomers were measured here. Only the Mi124, Mi133 and Mi134 species contained these linotrins. The greatest amount was found in Mi133 which had 6.3 ng/mg. Mi124 contained 3.5 ng/mg, which is approximately 10 times more than Mi134′s (0.3 ng/mg) (Supplementary Table S2).

- Non enzymatic profile

Phytoprostanes (PhytoPs) and phytofuranes (PhytoFs) are formed from non-enzymatic oxidation of ALA in plants [46].

**Figure 6:**
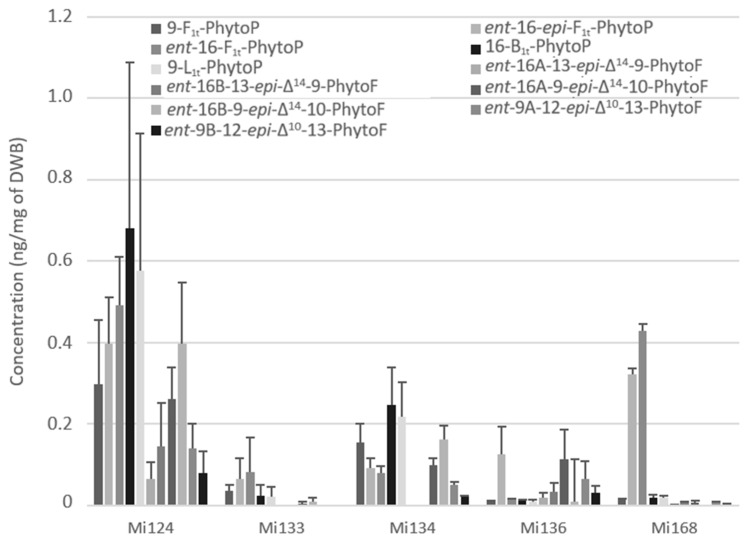
Non-enzymatic oxygenated metabolites of ALA profile in Mi124, Mi133, Mi134, Mi136 and Mi168 expressed in ng/mg (*n* = 3).

Figure 6 showed the presence of five PhytoPs and six PhytoFs in various concentrations in all five species, except for Mi134 and Mi136 where the *ent*-16A-13-*epi*-∆^14^-10-PhytoF and *ent*-16B-13-*epi*-∆^14^-10-PhytoF were not detected.

Regarding the total quantity, ALA derivatives were the predominant non-enzymatic oxylipins. The total concentration in marine microalgae was approximately 3.5 ng/mg for Mi124. In addition, Mi134 had the second-highest concentration with a concentration of 1.1 ng/mg of DWB (Figure 6). 

In decreasing order, we discovered that Mi168, Mi136, and Mi133 were the three lesser makers of ALA-oxidized derivatives, with 0.8 ng/mg, 0.6 ng/mg, and 0.2 ng/mg, respectively.

Interestingly, in Mi124 (2.4 ng/mg) and in Mi134 (0.8 ng/mg), the sum of PhytoFs concentration is two times higher than the sum of PhytoPs concentration (Figure 6). However, Mi133 and Mi168 did not follow the same upwards trend. In fact, PhytoPs were 15 times more abundant than PhytoFs in Mi133 and Mi168 (0.3 ng and 0.8 ng/mg, respectively). Only Mi136 displayed the opposite tendency, having four times lower PhytoPs (0.2 ng/mg) than PhytoFs.

#### 2.2.2. Derivatives of EPA

- Enzymatic profile

EPA is a suitable substrate for COX, LOX and CYP enzymes. Among all the EPA metabolites, only 18-hydroxyeicosapentaenoic acid (18-HEPE), a resolvin E-series precursor, was found in these marine matrices.

Regarding the PUFAs profile, Mi133, Mi136 and Mi168, algae which contain mainly EPA, produced 18-HEPE at a higher concentration than the other species. The level of this COX derivative was 78.6 ng/mg for Mi136 and 40.0 ng/mg for Mi133 and Mi168. Mi124 and Mi134 produced less than 2.0 ng/mg of 18-HEPE (Supplementary Table S2).

- Non-enzymatic profile

**Figure 7:**
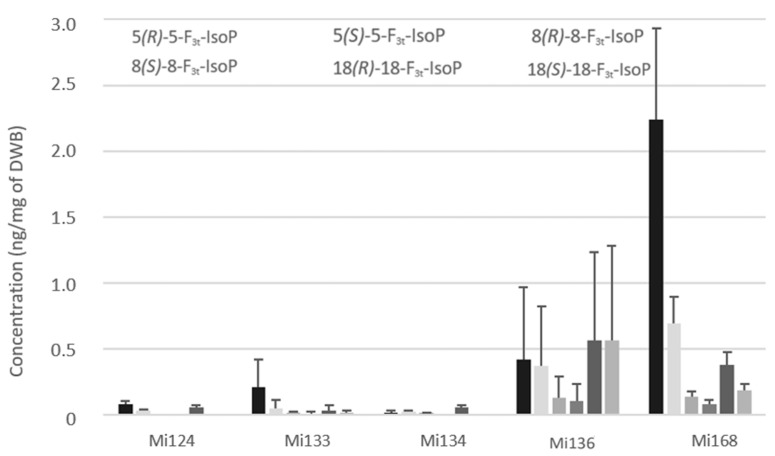
Non-enzymatic oxygenated metabolites of EPA profile in Mi124, Mi133, Mi134, Mi136 and Mi168 expressed in ng/mg (*n* = 3).

Concerning the non-enzymatic oxidation, the oxidized metabolites of looked EPA are F_3_-type isoprostanes (F_3t_-IsoPs). Five structures have been detected in microalgae (Figure 7). 

Expectedly, as with enzymatic oxylipins, Mi168 and Mi136 stood out with the highest concentration compared to Mi124 and Mi134. The sum of F_3t_-IsoPs concentration ranged from less than 0.1 ng/mg (Mi124 and Mi134) and 0.3 ng/mg in Mi133 to 2.1 ng/mg in Mi136 and 3.7 ng/mg in Mi168 (Figure 7).

These isoprostanes are epimers and can be classified by pairs. The amount of 8*(RS)*-8-F_3t_-IsoP was therefore less than 18*(RS)*-18-F_3t_-IsoP for each species.

#### 2.2.3. Derivatives of DHA

- Enzymatic profile

**Figure 8:**
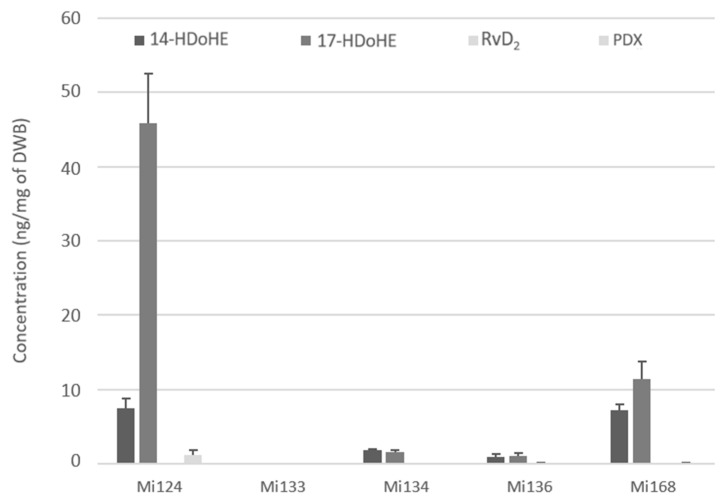
Enzymatic oxygenated metabolites of DHA profile in Mi124, Mi133, Mi134, Mi136 and Mi168 expressed in ng/mg (*n* = 3).

DHA can generate some docosanoids by LOX reactions such as resolvins and protectins. Except for Mi133, which contains none, marine microalgae have been shown to include two intermediates, a resolvin and/or a protectin (Figure 8).

These metabolites are detected with a total of 54.6 ng/mg for Mi124, followed by Mi168 whose concentration is three times lower (18.7 ng/mg). The concentration of Mi134 and Mi136 in LOX-docosanoids was 10 times lower than it was in Mi136 the others (less than 3.5 ng/mg) (Figure 8).

Each species had a 14-hydroperoxide intermediate coming from the 12-LOX pathway, with concentrations ranging from 0.9 ng to 7.4 ng/mg.

17-HDoHE, the second 15-LOX metabolite, was likewise found in each species, but at varying concentrations ranging from 1.1 ng to 45.9 ng/mg. This metabolite is a precursor of protectins, especially PDX, which was detected in Mi124 and Mi168 (1.3 ng/mg and 0.1 ng/mg).

In addition, one single resolvin of D-series, RvD_2_, was found in modest quantities in Mi136 (150 pg/mg) (Figure 8).

- Non-enzymatic profile

**Figure 9:**
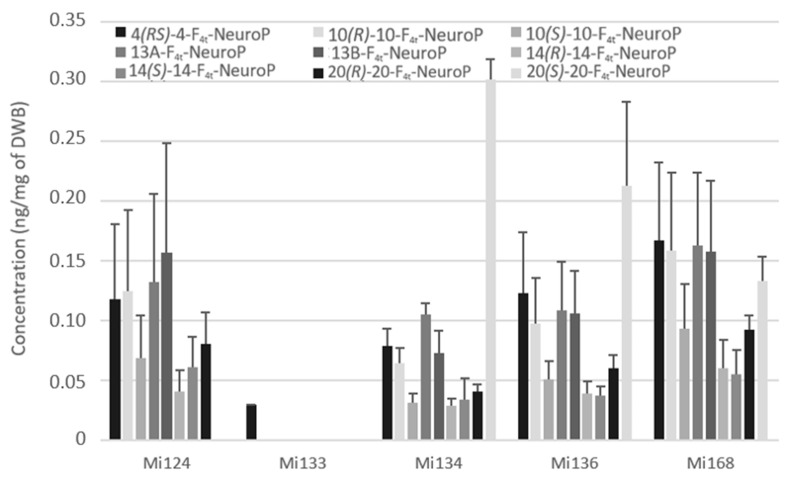
Non-enzymatic oxygenated metabolites of DHA profile in Mi124, Mi133, Mi134, Mi136 and Mi168 expressed in ng/mg (*n* = 3).

Non-enzymatic oxygenation of DHA induces the formation of neuroprostanes (F_4t_-NeuroPs). Figure 9 highlights that the overall composition of different species is similar.

Only the 4(*RS*)-F_4t_-NeuroP, which was detected at less than 0.1 ng/mg (29 pg/mg of DWB), was present in Mi133 which displayed in accordance with its production of trace levels of DHA (Figure 9).

By contrast, Mi168 displayed an amount of 1.3 ng/mg in accordance with its main EPA/DHA profile. A wide variety of these metabolites allowed Mi124, Mi136, and Mi134 to achieve around 0.8 ng/mg.

## 3. Discussion

In the present study, five microalgae categorized in four different phyla were shown to synthesize a wide range of omega-3 and -6 derivatives.

Among the species already produced and cultivated at the Microphyt strain, five species were chosen due to their specific omega-3 PUFA profile. Analysis of a species that is rich in ALA (Mi133, Mi134), EPA (Mi136), DHA (Mi124) and EPA/DHA (Mi168) was performed. This diversity of PUFAs detected may illustrate the heterogeneity in oxidized products formed.

Until now, many studies have focused on the generation of *n*-3 PUFAs in microalgae, such as EPA and DHA [46], but few studies have emphasized the profile of oxygenated metabolites [44,45].

Here, we present a qualitative and quantitative profile of five marine microalgae’s non-enzymatic and enzymatic oxylipins under controlled culture conditions in 10 L. To our knowledge, this is the first broad oxylipin profile reported in microalgae, combining a fingerprint in enzymatic and non-enzymatic oxygenated metabolites in a 10 L system. Our findings extend the repertoire of oxylipins that are naturally found in microalgae. Twenty-four enzymatic metabolites and 33 non-enzymatic oxylipins were detected. The five microalgae explored have demonstrated a high capacity to synthesize a variety of oxidized derivatives, including both non-enzymatic and enzymatic oxylipins. The various profiles obtained showed variability in terms of metabolites concentration and signature that are specifically present in certain species. Each species has exhibited its own oxylipins profile with varying degrees of diversity.

Linotrins (enzymatically oxidized derivatives) were studied in microalgae for the first time, and larger concentrations were detected in Mi133. Linotrins are one of the target metabolites with biological activity that exhibits a reduction in microglia inflammation [23].

Furthermore, Mi133 and Mi134, which have an ALA abundant profile, do not appear to be the most attractive species to exploit for their oxylipin’s biological activities based on our results.

DHA derivative concentrations were relatively low in Mi133. This species has increased concentrations of ARA-derived compounds, such as H-ETEs, TXB_2_, PGs which often exert pro-inflammatory effects [47,48]. The lowest amount of oxylipins is also detected in Mi134.

Mi124, which had a DHA profile, displayed a high concentration of 17-HDoHE as well as the presence of PDX and the larger amounts of ALA that had been non-enzymatically oxidized. PhytoP/Fs and DHA-derivatives play a role in immunomodulatory and anti-inflammatory mechanisms [49,50,51].

Mi136, which had an EPA profile, is notable for having a high concentration of 18-HEPE (an EPA enzymatic derivative) which can inhibit myocardial fibroblasts proinflammatory activation [52].

A rich DHA derivative and the majority of EPA non-enzymatic derivative fingerprints were shown by the Mi168, which had EPA and DHA profiles. The properties of these detected compounds are well recognized. Oxylipins derived from EPA and DHA exhibit strong anti-inflammatory effects [10,35,51].

We have noticed a correlation between the quantity of PUFAs present in microalgae and their capacity to be converted into oxylipins. Indeed, Mi133 exhibits the highest concentration of linotrins enzymatic derivatives; greater levels of PUFA are also found in this same microalga. Likewise, the most abundant PUFA in Mi136, EPA, is also the source of high levels of enzymatic oxylipins. Similar circumstances apply to Mi124 and Mi168, exhibiting an abundant DHA profile, which show the highest amount of enzymatically oxygenated metabolites of DHA.

The five species tested showed a particular pattern. Enzymatic oxylipins, which were approximately ten times more concentrated than non-enzymatic derivatives, were the most prevalent metabolites in microalgae. These microalgae fingerprints are, for now, the most complete in terms of oxylipins within microalgae. These results were obtained from biologically distinctive samples to have a closer view of reality. The microalgae production depends on several factors, such as culture and biological models. It was interesting to see the oxylipin variation among biological triplicates from a semi-continuous culture. 

A highly sensitive technique was used to detect the metabolites. In fact, the limits of detection (LODs) for enzymatic metabolites ranged from 0.01 ng/mL to 15.6 ng/mL while the limits of quantification (LOQs) ranged from 0.03 ng/mL to 32 ng/mL. Additionally, the NEO-PUFAs LOD and LOQ values varied from 0.16 ng/g to 0.63 ng/g and from 0.16 ng/g to 1.25 pg/g, respectively for detection and quantification. Moreover, a targeted lipidomic method was used to study 69 oxygenated derivatives utilizing 26 enzymatic standards and 43 non-enzymatic standards. Thus, we are able to detect 57 of them, 24 enzymatic and 33 non-enzymatic metabolites, in various concentrations ranging from 0.1 ng/mg for the lowest to 233 ng/mg for the highest concentration. 

Our knowledge of the oxidized metabolites and the chemical compounds that each microalga can produce has increased as a result of this work. Although oxylipins have interesting biological effects on humans, we know very little about their role in microalgae. Among some hypotheses, including antipredator, antibacterial, infochemical or allelochemical, a recent study demonstrated the bactericidal effect of oxidized EPA and DHA derivatives, the HEPEs [53]. In this study, an infected *Bacillariophyte* released oxylipins, including HEPEs in higher concentration, resolvins E, HDHAs and HETEs. This knowledge can help us to focus our cultures of specific microalgae according to the type of metabolites that are of interest.

The abundant oxylipins biosynthesis in microalgae showed a promising biological activity. Some research groups are interested in the anti-inflammatory potential of microalgal-derived oxylipins [54,55,56]. The interest in learning more about these marine sources of bioactive oxidized lipids with potent anti-inflammatory effects and health benefits was highlighted by the tests conducted herein. Therefore, microalgae can become new sustainable alternatives for human health.

## 4. Materials and Methods

### 4.1. The Microalgal Species and Culture Conditions

According to the diversity of their PUFAs, five different confidential species of microalgae were selected, with each having a different predominant PUFA. They include two *Haptophytes* “Mi124” (with DHA, the most abundant PUFA) and “Mi168” (with EPA/DHA predominant PUFA), a *Rhodophyte* “Mi133” (with ALA predominant PUFA), a *Chlorophyte* “Mi134” (with ALA predominant PUFA) and a *Bacillariophyte* “Mi136” (with EPA, the most predominant PUFA). All five species presented a varied PUFA profile.

In this study, marine microalgae were cultivated in biological triplicate in 10 L photobioreactors (PBR) at Microphyt manufacturing facility (Baillargues, France). A medium based on f/2 medium (Guillard and Ryther 1962) [57] with artificial sea water (15 g/L) was used to grow cells under optimal conditions according to their productivity. PBRs were under control temperature (24 °C). The pH of the medium was between 7.5 and 8, and the incoming air was enriched in CO_2_ (3%) with an average flowrate of 3 L/h. The light intensity (50 µmol.m^−2^.s^−1^) was constant throughout the growth provided by daylight LEDs under continuous 24/0 lighting.

Cultures were conducted in a semi-continuous process, beginning with the inoculation of a sterile medium, followed by a partial harvest (3/4) in the middle of the exponential growth phase, then the addition of a new sterile medium.

Cells were harvested by centrifugation at 6000 rpm (Rotixa 500 RS, Hettich, Germany) and stored frozen at −20 °C until analysis.

### 4.2. Non Enzymatic Oxylipins

#### 4.2.1. Chemicals and Reagents

Commercially available isoprostanoid standards (2,3-*dinor*-15-F_2t_-IsoP) and the internal standard (ISTD) D4-15-F_2t_-IsoP were purchased from Cayman Chemicals. Other internal standards (d4-10-F_4t_-NeuroP, C19-16-F_1t_-PhytoP and C21-15-F_2t_-IsoP) and isoprostanoid standards were synthesized according to the previous procedures including phytoprostanoids (9-F_1t_-PhytoP, *ent*-16-*epi*-16-F_1t_-PhytoP, *ent*-16-F_1t_-PhytoP, 9-L_1t_-PhytoP) and phytofuranes (*ent*-16*A*-13-*epi*-ST-∆^14^-9-PhytoF, *ent*-16*B*-13-*epi*-ST-∆^14^-9-PhytoF, *ent*-16*A*-9-*epi*-ST-∆^14^-10-PhytoF, *ent*-16*B*-9-*epi*-ST-∆^14^-10-PhytoF, *ent*-9*A*-12-*epi*-ST-∆^10^-13-PhytoF and *ent*-9*B*-12-*epi*-ST-∆^10^-13-PhytoF) obtained from the oxidation of alpha linolenic acid, ALA, C18:3 *n*-3; isoprostanoids derived from arachidonic acid, ARA, C20:4 *n*-6 (15-A_2t_-IsoP, 5-F_2c_-IsoP, 5-*epi*-5-F_2t_-IsoP, 5-F_2t_-IsoP); isoprostanoids derived from ecosapentaenoic acid acid EPA, C20:5 *n*-3 (5*(S)*-5-F_3t_-IsoP, 8*(R)*-8-F_3t_-IsoP, 8*(S)*-8-F_3t_-IsoP, 18*(R)*-18-F_3t_-IsoP and 18*(S)*-18-F_3t_-IsoP); and neuroprostanoids (4*(RS)*-4-F_4t_-NeuroP, 10*(R)*-10-F_4t_-NeuroP, 10*(S)*-10-F_4t_-NeuroP, 13*(A)*-13-F_4t_-NeuroP, 13*(B)*-13-F_4t_-NeuroP, 14*(R)*-14-F_4t_-NeuroP, 14*(S)*-14-F_4t_-NeuroP, and 20*(R)*-20-F_4t_-NeuroP) from docosahexaenoic acid, DHA, C22:6 *n*-3 and the last one, 4*(RS)*-4-F_3t_-NeuroP, coming from the oxidation of docopentaenoic acid, DPA C22:5 *n*-3. “A” and “B” designate the R or S configuration, but these are not determined.

Acetonitrile (ACN, LC-MS grade), methanol (MeOH, LC-MS grade), chloroform (CHCl_3_, HPLC grade) and water that has been purified using a milliQ system (H_2_O, LC-MS grade), were all obtained from Fisher Scientific.

Hexane (HPLC grade), absolute ethanol (EtOH, HPLC grade), formic and acetic acids (HCOOH and AcOH, HPLC grade) and butylated hydroxytoluene (BHT) were purchased from Sigma Aldrich.

Ethyl acetate (EtOAc) (>99.8%) and ammonia (NH_3_, 28%) were purchased from VWR.

Solid phase extraction cartridges (Oasis^®^ MAX Cartridge, 60 mg) were obtained from Waters. 

#### 4.2.2. Algal Sample Preparation

For this part of the analysis, the non-enzymatic oxylipins were extracted using a protocol that was previously published in prior work on marine macroalgae [40] with some modifications. 

The analysis was performed on fresh biomass, which means that the harvested biomass has not been dried. 

For the extraction, 100 mg of fresh biomass were placed in lysing matrix tubes (lysing matrix D, MP Biochemicals, Illkirch, France) with 25 µL of BHT (butylated hydroxytoluene 1% in water), 1 mL of MeOH and 4 µL of ISTD mix (1 ng/µL). The sample was then grinded using a FastPrep-24 (MP Biochemicals) at a speed of 6.5 m/s for 30 s.

The mixture was transferred into a 15 mL centrifuge tube with 1 mL of MeOH and 1.5 mL of phosphate buffer (50 mM, pH 2.1, prepared with NaH_2_PO_4_ and H_3_P O_4_) saturated in NaCl. The tubes were shaken for 30 min at room temperature, using a vertical rotator (PTR-60, Grant Instruments, Cambridge) at 360° vertical rotation for 45 s and 90° rotation for 15 s. The samples were then centrifuged at 5000 rpm for 5 min at room temperature. The supernatant was collected into another 15 mL centrifuge tube with 4 mL of cold chloroform and was stirred with a vortex mixer for 30 s. Then the mixture was centrifuged for 5 min at 4 °C at 2000 rpm.

The lower organic phase was collected in Pyrex tubes and was then dried under N_2_ for an average of 1 h at 40 °C in a dry bath. To extract lipid fraction, the dried extract was hydrolyzed by adding 950 µL of 1 M KOH and was incubated at 40 °C for 30 min with a vertical rotator (100 rpm). The mixture was added with 1 mL of 40 mM formic acid prior to starting the solid phase extraction on the automated sample processing. 

After that, samples were loaded on pre-conditioned Oasis mixed polymeric sorbent cartridges (Oasis MAX Cartridge, 60 mg, Waters). The undesired compound was then eliminated using 1.5 mL of NH_3_ 2% (*v*/*v*), 1.5 mL of MeOH/20 mM formic acid (30:70; *v*/*v*), 1.5 mL of hexane, and 1.5 mL of hexane/ethyl acetate (70:30; *v*/*v*). Finally, isoprostanoids were eluted by adding 2 × 1 mL of a mixture of Hexane/EtOH/Acetic acid (70:29.4:0.6; *v*/*v*/*v*). The samples were dried in a dry bath at 40 °C for an average 1 h under a nitrogen flow.

The dried extracts were reconstituted with 100 µL of mobile phase solvents (H_2_O/ACN; 83:17; *v*/*v*) and then filtered in 0.45 µm Eppendorf (Nanosep Centrifugal Devices) with a centrifugation at 1000 rpm for 1 min at room temperature.

The analysis was completed by injecting 5 µL of the extract into the micro-LC-MS/MS 5500 QTrap system, which uses high-performance liquid chromatography coupled to tandem mass spectrometry.

#### 4.2.3. Micro-LC-MS/MS Analysis

An Eksigent micro- High performance liquid chromatography (HPLC) 200 Plus (Sciex Applied Biosystems, Framingham, MA, USA) equipped with CTC Analytics AG (Zwingen, Switzerland) was used and all analyses were carried out on a HALO C18 analytical column (100 × 0.5 mm, 2.7 µm; Eksigent Technologies, CA, USA) maintained at 40 °C. 

The mobile phases consisted of a binary gradient of H_2_O with 0.1% (*v*/*v*) HCOOH (solvent A) and ACN with 0.1% (*v*/*v*) HCOOH (solvent B) with a flow rate of 0.03 mL.min^−1^ and an injection volume of 5 µL. The elution gradient was as follows: 17% B at 0 min, 17% B at 2.6 min, 21% B at 2.85 min, 25% B at 7.3 min, 28.5% B at 8.8 min; 33.3% B at 11 min; 40% B at 15 min, 95% B at 16.5 min for 1.5 min.

Using an electrospray ionization (ESI) in negative mode, mass spectrometry analyses were performed on an AB Sciex QTRAP 5500 (Sciex Applied Biosystems, ON, Canada). The source is maintained at −4.5 kV, and nitrogen flow serves as curtain gas at 30 psi and a nebulization assist at 20 psi, at room temperature.

In order to analyze the targeted compounds with a detection window of 90 s, the monitoring of the ionic fragmentation products of each deprotonated analyte [M-H]- molecule was carried out in Multiple Ion Monitoring (MRM) detection mode using nitrogen as the collision gas. Two transitions for quantification (T1) and specification (T2) were pre-determined by MS/MS analysis of standards. 

LC-MS/MS data acquisition was performed using the Analyst^®^ software (Sciex Applied Biosystems), which drives the mass spectrometer. The peak integration and quantification of analytes were processed by MultiQuant 3.0 software (Sciex Applied Biosystems).

#### 4.2.4. Validation of Sample Preparation

The efficiency of sample preparation and the validity of microalgal isoprostanoid profiles were assessed using the global process efficiency (PE), matrix effect (ME) and extraction recovery (ER) [58,59,60] (tables in Supplementary Materials).

In brief, four different sets were prepared at high concentrations for each compound (200 ng/mL).

The first (set 1) was obtained with a spike of 5 µL of an isoprostanoid mixture (4 µg/mL) into 100 mg of fresh microalgal biomass in biological triplicate for each concentration at the beginning of the extraction process described above.

The identical procedure as set 1 was used for the second set (set 2) but the microalgal samples were spiked after extraction.

The last two sets consisted of extraction without microalgal matrix and spiked with 5 µL of the mixture before the extraction step (set 3) and prepared in 100 µL of mobile phase H_2_O/ACN (83:17; *v*/*v*) spiked with 5 µL of the mixture (set 4).

PE was calculated as a percentage of set 1 peak areas (spike before extraction) to the set 3 peak areas (neat extraction solution). The ME was evaluated by comparing the set 2 peak areas (spike after extraction) and the set 3 peak areas (neat extraction solution).

Finally, the ER was expressed as the ratio of the peak areas of set 1 (spike before extraction) to that of set 2 (spike after extraction).

### 4.3. Enzymatic Oxylipins

#### 4.3.1. Algal Sample Preparation

Internal Standards (ISTD): LxA4-d5, LTB4-d4, 5-HETE-d8

The enzymatic oxylipins at the MetaToul lipidomic facility were quantified using a similar protocol to that described by P. Le Faouder [61] was applied to quantify the enzymatic oxylipins at the MetaToul lipidomic facility.

Briefly, 25 mg of fresh biomass and 500 µL of HBSS were crushed with a FastPrep^®^ Instrument (MP Biomedicals, LLC, Santa Ana, CA, USA). The homogenate samples were mixed with 260 µL of cold MeOH and 40 µL of ISTD solution (50 ng/mL) and then centrifuged at 5000 rpm for 15 min at 4 °C.

Supernatants were collected, completed to 2 mL of H_2_O and submitted to solid phase extraction using HRX-50 mg 96-well clusters (Macherey Nagel, Hoerd, France). 

The sample was loaded after conditioning the plate with 1 mL of MeOH and 1 mL of H_2_O/MeOH (90:10, *v*/*v*). At this step, the plate was washed with 1 mL of H_2_O/MeOH (90:10, *v*/*v*), and the lipid mediators were eluted with 1 mL of MeOH. The samples were evaporated in a dry bath at 40 °C under a nitrogen flow.

The dried extracts were dissolved in 10 µL of MeOH and 5 μL of the resulting extract was injected and was analyzed by using a LC-MS/MS.

#### 4.3.2. LC-MS/MS Analysis

Eicosanoid analysis was performed using an HPLC Agilent 1290 Infinity on a ZorBAX SB-C18 analytical column (50 × 2.1 mm, 1.8 μm) (Agilent Technologies) maintained at 40 °C. The mobile phases were composed of two solvents: solvent A, H_2_O with 0.1% (*v*/*v*) HCOOH, and solvent B, ACN with 0.1% (*v*/*v*) HCOOH. The flow rate of the mobile phase was 0.35 mL.min^−1^, and the injection volume was 5 µL. The gradient of the elution was as follows: 0% B at 0 min, 85% B at 8.5 min, 100% B at 9.5 min for 1 min. 

The liquid chromatography was coupled to an Agilent 6460 triple quadrupole MS with an ESI source in negative mode. The monitoring of fragmentation was executed in Selection Reaction Monitoring (SRM) detection mode. Finally, peak detection, integration and quantitative analysis were obtained using the MassHunter Quantitative analysis software version B.09.00 (Agilent Technologies).

## 5. Conclusions

The five studied microalgae revealed a high diversity of lipid metabolites, including up to 57 oxylipins found in various amounts, with the most complete report displaying many oxidized lipids. It should be highlighted that the total enzymatic metabolites were predominant over non-enzymatic oxylipins in these marine matrices.

Taken together, these findings show the potential of microalgae as a new source of bioactive lipid mediators with specific profiles for each species. As reported by J.M. Galano, J. Roy and X. Geng, DHA-derived metabolites demonstrate several biological properties, including antiarrhythmic properties [36,62], protective effect on cardiomyocytes [37] and neuroprotective effects [63]. Therefore, microalgae appear to be potential candidates for cardiovascular diseases research.

Further work should focus on the culture conditions applied to guide its metabolism toward a more meaningful production of oxylipins as some studies have indicated [44,45]. Many applications can therefore be envisaged with microalgae and could valorize oxylipins on an industrial scale in the form of natural active ingredients to promote the reduction of inflammation in preventive medicine and to promote well-being.

## Data Availability

Not applicable.

## Supplementary Materials

The following are available online at https://www.mdpi.com/article/10.3390/md21030136/s1, Table S1, S2: Quantification of non-enzymatic and enzymatic oxygenated metabolites of omega-6 and omega-3 in Mi124, Mi133, Mi134, Mi136 and Mi168; Table S3–S7: The efficiency of sample preparation of Mi124, Mi133, Mi134, Mi136, Mi168 with the extraction recovery (ER), the matrix effect (ME) and the global process efficiency (PE).

## Abbreviations

PUFA	polyunsaturated fatty acid
LA	linoleic acid
AA	arachidonic acid
DPA	docosapentanoic acid
ALA	alpha-linolenic acid
EPA	eicosapentaenoic acid
DHA	docosahexaenoic acid
OS	oxidative stress
NEO-PUFAs	non-enzymatic oxidized PUFAs
ROS	reactive oxygenated species
COX	cyclooxygenase
LOX	lipoxygenase
CYP	cytochrome
SPM	specialized proresolving mediators
DWB	dry-weight biomass
ME	matrix effect
ER	extraction recovery
PE	process efficiency
HODEs	hydroxy-octadecadienoic acids
LXs	lipoxins
LTs	leukotrienes
H-ETEs	hydroxy-eicosatetraenoic acids
EETs	epoxyeicosatrienoic acids
PGs	prostagladines
TXBs	thromboxanes
HEPE	hydroxyeicosapentaenoic acid
HDoHE	hydroxydocosahexaenoic acid
IsoPs	isoprostanes
NeuroPs	neuroprostanes
PhytoPs	Phytoprostanes
PhytoFs	phytofuranes
