# Supplementary material for: Bioactive Oxylipins Profile in Marine Microalgae
Source: Mar Drugs. 2023 Feb 22;21(3):136. doi: 10.3390/md21030136 (PMC10051100; doi:10.3390/md21030136)
Supplement: Supplementary file 1 [file marinedrugs-21-00136-s001.zip › marinedrugs-2177954-supplementary.pdf]

marine drugs

MDPI

Supplementary Materials: Bioactive Oxylipins Profile in Marine Microalgae

All quantification of non-enzymatic and enzymatic oxygenated metabolites of omega-6 and 3 in Mi124, Mi133, Mi134, Mi136 and Mi168 are available as Supplementary Materials, Table S1 for oxygenated metabolites of omega-6 and Table S2 for oxygenated metabolites of omega-3. Data are mean ± sd (n = 3) expressed as ng/mg of dry weight biomass. ND for No Detected.

Table S1. Quantification of non-enzymatic and enzymatic oxygenated metabolites of omega-6 in Mi124, Mi133, Mi134, Mi136 and Mi168.

	Component Name	Mi124			Mi133			Mi134			Mi136			Mi168		
		Concentration (ng/mg)	±	SD	Concentration (ng/mg)	±	SD	Concentration (ng/mg)	±	SD	Concentration (ng/mg)	±	SD	Concentration (ng/mg)	±	SD
LA	9-HODE	37.10	±	14.68	22.00	±	4.86	1.71	±	0,92	9.17	±	3.88	0.03	±	0.10
	13-HODE	108.44	±	27.71	27.29	±	6.84	3.50	±	1.60	12.81	±	5.05	0.48	±	0.16
ARA	5-HETE	0.53	±	0.06	66.92	±	24.92	1.55	±	0.67	5.11	±	5.38	5.30	±	0.65
	8-HETE	0.14	±	0.03	41.50	±	11.29	1.28	±	0.37	3.36	±	3.28	2.95	±	0.68
	12-HETE	0.58	±	0.07	50.96	±	17.22	2.13	±	0.75	4.45	±	4.30	3.44	±	0.88
	15-HETE	0.75	±	0.09	53.72	±	13.91	0.22	±	0.09	1.00	±	0.87	0.99	±	0.05
	5,6-DiHETE	ND	±	ND	0.07	±	0.03	ND	±	ND	ND	±	ND	ND	±	ND
	5oxoETE	0.07	±	0,06	15.62	±	4.72	0.30	±	0.09	0.49	±	0.47	0.51	±	0.18
	5,6-EET	ND	±	ND	6.67	±	2.96	0.11	±	0.02	0.19	±	0.16	0.32	±	0.04
	11,12-EET	ND	±	ND	17.97	±	5.90	0.14	±	0.02	0.22	±	0.23	0.23	±	0.02
	14,15-EET	0.06	±	0.01	14.35	±	4.36	0.14	±	0.03	0.23	±	0.20	0.27	±	0.02
	LTB4	ND	±	ND	0.39	±	0.16	ND	±	ND	ND	±	ND	ND	±	ND
	TXB2	ND	±	ND	ND	±	ND	ND	±	ND	ND	±	ND	0.07	±	0.01
	PGE2	ND	±	ND	0.07	±	0.03	ND	±	ND	ND	±	ND	ND	±	ND
	PGF2α	ND	±	ND	0.34	±	0.19	ND	±	ND	ND	±	ND	ND	±	ND
	8-iso-PGA2	ND	±	ND	0.16	±	0.10	ND	±	ND	ND	±	ND	ND	±	ND
	15-d-PGJ2	0.70	±	0.68	0.31	±	0.40	0.56	±	0.16	1.09	±	0.69	2.03	±	1.00
	PGD2	ND	±	ND	0.45	±	0.23	ND	±	ND	ND	±	ND	ND	±	ND
	15-A_{2t}-IsoP	0.07	±	0.02	0.03	±	0.02	ND	±	ND	0.18	±	0.07	0.25	±	0.06
	5(RS)-5-F_{2c}-IsoP	0.01	±	< 0.01	0.04	±	0.02	0.10	±	0.04	0.42	±	0.30	0.17	±	0.05
	5(RS)-5-F_{2t}-IsoP	0.01	±	< 0.01	0.03	±	0.01	0.06	±	0.02	0.24	±	0.12	0.10	±	0.03
	15-epi-F_{2t}-IsoP	ND	±	ND	0.01	±	< 0.01	0.02	±	0.01	0.08	±	0.04	0.03	±	0.01
	15-F_{2t}-IsoP	ND	±	ND	< 0.01	±	< 0.01	0.01	±	0.01	0.05	±	0.02	0.02	±	< 0.01
	2.3-dinor-15-F_{2t}-IsoP	0.01	±	0.01	ND	±	ND	0.01	±	< 0.01	0.01	±	< 0.01	ND	±	ND
DPA	4-F_{3t}-NeuroP	0.04	±	0.02	0.01	±	0.01	0.01	±	< 0.01	0.02	±	< 0.01	0.22	±	0.09

Table S2. Quantification of non-enzymatic and enzymatic oxygenated metabolites of omega-3 in Mi124, Mi133, Mi134, Mi136 and Mi168.

	Component Name	Mi124			Mi133			Mi134			Mi136			Mi168		
		Concentration (ng/mg)	±	SD	Concentration (ng/mg)	±	SD	Concentration (ng/mg)	±	SD	Concentration (ng/mg)	±	SD	Concentration (ng/mg)	±	SD
ALA	9(R),16(R,S)-linotrins	3.52	±	0.92	6.29	±	2.80	0.03	±	0.01	ND	±	ND	ND	±	ND
	9-F_{1t}-PhytoP	0.30	±	0.16	0.04	±	0.01	0.16	±	0.05	0.01	±	< 0.01	0.01	±	< 0.01
	ent-16-F_{1t}-PhytoP	0.49	±	0.12	0.08	±	0.08	0.08	±	0.02	0.01	±	< 0.01	0.43	±	0.02
	ent-16-epi-16-F_{1t}-PhytoP	0.40	±	0.11	0.07	±	0.05	0.09	±	0.02	0.13	±	0.10	0.32	±	0.02
	16B_{1t}-PhytoP	0.68	±	0.41	0.02	±	0.03	0.25	±	0.09	0.01	±	< 0.01	0.02	±	< 0.01
	9L_{1t}-PhytoP	0.58	±	0.34	0.02	±	0.02	0.22	±	0.09	0.01	±	< 0.01	0.02	±	< 0.01
	ent-16(A)-13-epi-ST-Δ^{14}-9-PhytoF	0.06	±	0.04	ND	±	ND	ND	±	ND	0.02	±	0.01	< 0.01	±	< 0.01
	ent-16(B)-13-epi-ST-Δ^{14}-9-PhytoF	0.15	±	0.10	ND	±	ND	ND	±	ND	0.03	±	0.02	< 0.01	±	< 0.01
	ent-16(A)-9-epi-ST-Δ^{14}-10-PhytoF	0.26	±	0.08	< 0.01	±	< 0.01	0.10	±	0.02	0.11	±	0.07	0.01	±	< 0.01
	ent-16(B)-9-epi-ST-Δ^{14}-10-PhytoF	0.40	±	0.15	0.01	±	0.01	0.17	±	0.03	0.16	±	0.10	0.01	±	< 0.01
	ent-9(A)-12-epi-ST-Δ^{10}-13-PhytoF	0.14	±	0.06	ND	±	ND	0.05	±	0.01	0.07	±	0.04	0.01	±	< 0.01
	ent-9(B)-12-epi-ST-Δ^{10}-13-PhytoF	0.08	±	0.05	ND	±	ND	0.02	±	< 0.01	0.03	±	0.02	< 0.01	±	< 0.01
EPA	18-HEPE	2.28	±	0.45	39.47	±	14.65	0.30	±	0.14	78.61	±	3.10	39.96	±	3.07
	5(R)-5-F_{3t}-IsoP	0.08	±	0.03	0.20	±	0.21	0.01	±	0.01	2.59	±	3.52	2.24	±	0.70
	5(S)-5-F_{3t}-IsoP	0.03	±	0.01	0.05	±	0.06	0.02	±	< 0.01	0.37	±	0.45	0.69	±	0.20
	8(R)-8-F_{3t}-IsoP	ND	±	ND	0.01	±	0.01	0.01	±	< 0.01	0.13	±	0.16	0.14	±	0.04
	8(S)-8-F_{3t}-IsoP	ND	±	ND	0.01	±	0.01	ND	±	ND	0.10	±	0.13	0.08	±	0.03
	18(R)-18-F_{3t}-IsoP	0.05	±	0.02	0.03	±	0.04	0.05	±	0.02	0.56	±	0.67	0.38	±	0.01
	18(S)-18-F_{3t}-IsoP	ND	±	ND	0.01	±	0,02	ND	±	ND	0.56	±	0.72	0.18	±	0.05
DHA	14-HDoHE	7.44	±	1.34	ND	±	ND	1.90	±	0.12	0.95	±	0.42	7.22	±	0.74
	17-HDoHE	45.86	±	6.62	ND	±	ND	1.58	±	0.22	1.12	±	0.30	11.41	±	2.33
	RvD_2	ND	±	ND	ND	±	ND	ND	±	ND	0.15	±	0.04	ND	±	ND
	PDx	1.26	±	0.62	ND	±	ND	ND	±	ND	ND	±	ND	0.10	±	0.03
	4(RS)-4-F_{4t}-NeuroP	0.12	±	0.06	0.01	±	0.01	0.08	±	0.01	0.12	±	0.05	0.17	±	0.07
	10(R)-F_{4t}-NeuroP	0.12	±	0.07	ND	±	ND	0.06	±	0.01	0.10	±	0.04	0.16	±	0.06
	10(S)-F_{4t}-NeuroP	0.07	±	0.04	ND	±	ND	0.03	±	0.01	0.05	±	0.02	0.09	±	0.04
	13(A)-13-F_{4t}-NeuroP	0.13	±	0.08	ND	±	ND	0.10	±	0.01	0.11	±	0.04	0.16	±	0.06
	13(B)-13-F_{4t}-NeuroP	0.16	±	0.09	ND	±	ND	0.07	±	0.02	0.11	±	0.03	0.16	±	0.06
	14(R)-14-F_{4t}-NeuroP	0.04	±	0.02	ND	±	ND	0.03	±	0.01	0.04	±	0.01	0.06	±	0.02
	14(S)-14-F_{4t}-NeuroP	0.06	±	0.03	ND	±	ND	0.03	±	0.02	0.04	±	0.01	0.05	±	0.02
	20(R)-20-F_{4t}-NeuroP	0.08	±	0.03	ND	±	ND	0.04	±	0.01	0.06	±	0.01	0.09	±	0.01
	20(S)-20-F_{4t}-NeuroP	ND	±	ND	ND	±	ND	0.30	±	0.02	0.21	±	0.07	0.13	±	0.02

The measured ER, ME and PE at high concentrations are available as Supplementary Materials, Table S3 for Mi124, Table S4 for Mi133, Table S5 for Mi134, Table S6 for Mi136 and Table S7 for Mi168.

Table S3. The efficiency of sample preparation of Mi124 with the extraction recovery (ER), the matrix effect (ME) and the global process efficiency (PE).

Compound	Concentration (ng/mL)	Extraction recovery (ER)	±	SD	Matrix Effect (ME)	±	SD	Process Efficiency (PE)	±	SD
D4-10(*R*)-10F_{4t} NeuroP	40	38.91%	±	17.37%	59.81%	±	6.78%	23.27%	±	17.02%
D4-15-F_{2t}-IsoP	40	35.29%	±	15.60%	70.07%	±	4.78%	24.73%	±	16.71%
C21 15F_{2t} IsoP	40	41.01%	±	17.79%	62.12%	±	6.22%	25.47%	±	17.77%
C19 16-F_{1t}-PhytoP	40	38.13%	±	17.93%	62.67%	±	6.56%	23.90%	±	15.96%
15-A_{2t}-IsoP	200	0.88%	±	28.40%	50.28%	±	3.23%	0.44%	±	26.20%
5(*RS*)-5-F_{2c}-IsoP	200	54.64%	±	9.01%	73.44%	±	4.56%	40.12%	±	5.06%
5(*RS*)-5-F_{2t}-IsoP	200	64.03%	±	8.87%	71.78%	±	6.17%	45.96%	±	3.70%
2.3-*dinor*-15-F_{2t}-IsoP	200	52.43%	±	13.53%	80.73%	±	9.98%	42.33%	±	5.29%
4-F_{3t}-NeuroP	200	58.05%	±	13.36%	58.37%	±	4.68%	33.89%	±	9.36%
9-F_{1t}-PhytoP	200	45.92%	±	9.78%	80.86%	±	5.13%	37.13%	±	6.25%
ent-16-*epi*-16-F_{1t}-PhytoP	200	48.77%	±	8.90%	76.89%	±	4.12%	37.50%	±	6.56%
ent-16-F_{1t}-PhytoP	200	46.65%	±	12.63%	49.03%	±	5.71%	22.87%	±	9.40%
16B_{1t}-PhytoP	200	51.80%	±	10.72%	65.66%	±	4.42%	34.01%	±	6.82%
9L_{1t}-PhytoP	200	79.74%	±	9.64%	72.21%	±	4.42%	57.58%	±	6.55%
ent-16(*A*)-13-*epi*-ST-Δ^{14}-9-PhytoF	200	45.81%	±	7.02%	82.34%	±	3.94%	37.72%	±	5.58%
ent-16(*B*)-13-*epi*-ST-Δ^{14}-9-PhytoF	200	43.82%	±	8.77%	83.15%	±	4.34%	36.43%	±	8.08%
ent-16(*A*)-9-*epi*-ST-Δ^{14}-10-PhytoF	200	47.71%	±	12.22%	74.13%	±	4.88%	35.37%	±	9.79%
ent-16(*B*)-9-*epi*-ST-Δ^{14}-10-PhytoF	200	49.56%	±	7.57%	17.60%	±	4.75%	8.72%	±	6.09%
ent-9(*A*)-12-*epi*-ST-Δ^{10}-13-PhytoF	200	44.02%	±	8.72%	80.75%	±	3.19%	35.55%	±	7.13%
ent-9(*B*)-12-*epi*-ST-Δ^{10}-13-PhytoF	200	44.30%	±	8.73%	81.71%	±	5.88%	36.20%	±	7.07%
5(*R*)-5-F_{3t}-IsoP	200	95.73%	±	10.87%	42.29%	±	9.38%	40.48%	±	8.55%
5(*S*)-5-F_{3t}-IsoP	200	64.96%	±	10.33%	73.36%	±	6.11%	47.65%	±	7.44%
18(*R*)-18-F_{3t}-IsoP	200	52.56%	±	8.17%	87.09%	±	4.59%	45.77%	±	6.43%
4(*RS*)-4-F_{4t}-NeuroP	200	52.65%	±	12.20%	68.02%	±	4.61%	35.81%	±	8.28%
10(*R*)-F_{4t}-NeuroP	200	53.23%	±	10.94%	73.78%	±	5.44%	39.28%	±	6.88%
10(*S*)-F_{4t}-NeuroP	200	51.27%	±	12.51%	55.04%	±	7.90%	28.22%	±	7.58%
13(*A*)-13-F_{4t}-NeuroP	200	53.43%	±	9.87%	73.35%	±	5.35%	39.19%	±	6.60%
13(*B*)-13-F_{4t}-NeuroP	200	50.37%	±	13.46%	56.89%	±	5.81%	28.65%	±	8.51%
14(*S*)-14-F_{4t}-NeuroP	200	50.56%	±	11.36%	64.50%	±	7.07%	32.61%	±	6.43%
14(*S*)-14-F_{4t}-NeuroP	200	50.15%	±	10.07%	61.38%	±	5.65%	30.78%	±	4.94%
20(*R*)-20-F_{4t}-NeuroP	200	49.07%	±	11.22%	71.90%	±	7.18%	35.28%	±	8.37%

Table S4. The efficiency of sample preparation of Mi133 with the extraction recovery (ER), the matrix effect (ME) and the global process efficiency (PE).

Compound	Concentration (ng/mL)	Extraction recovery (ER)	±	SD	Matrix Effect (ME)	±	SD	Process Efficiency (PE)	±	SD
D4-10(*R*)-10F_{4t} NeuroP	40	80.67%	±	7.55%	71.09%	±	7.04%	57.35%	±	6.93%
D4-15-F_{2t}-IsoP	40	74.52%	±	6.75%	71.43%	±	6.22%	53.23%	±	6.42%
C21 15F_{2t} IsoP	40	87.92%	±	6.74%	66.22%	±	5.82%	58.22%	±	7.12%
C19 16-F_{1t}-PhytoP	40	76.38%	±	5.51%	79.48%	±	4.56%	60.71%	±	5.53%
15-A_{2t}-IsoP	200	-23.79%	±	8.76%	40.82%	±	14.24%	-9.71%	±	0.01%
5(*RS*)-5-F_{2c}-IsoP	200	88.89%	±	9.55%	104.26%	±	6.24%	92.68%	±	3.91%
5(*RS*)-5-F_{2t}-IsoP	200	89.63%	±	5.22%	58.16%	±	4.95%	52.13%	±	1.28%
15-*epi*-F_{2t}-IsoP	200	147.08%	±	4.54%	41.06%	±	2.18%	60.39%	±	3.86%
2.3-*dinor*-15-F_{2t}-IsoP	200	65.05%	±	1.87%	86.64%	±	2.44%	56.36%	±	1.17%
4-F_{3t}-NeuroP	200	81.56%	±	6.64%	92.24%	±	7.54%	50.52%	±	3.54%
9-F_{1t}-PhytoP	200	65.83%	±	2.33%	87.22%	±	2.44%	57.42%	±	1.50%
ent-16-F_{1t}-PhytoP	200	70.85%	±	3.35%	78.60%	±	3.20%	55.69%	±	2.63%
ent-16-*epi*-16-F_{1t}-PhytoP	200	66.80%	±	2.81%	81.03%	±	2.45%	54.13%	±	2.13%
16B_{1t}-PhytoP	200	75.58%	±	3.89%	77.32%	±	2.56%	58.44%	±	1.85%
9L_{1t}-PhytoP	200	103.72%	±	2.98%	79.65%	±	2.42%	82.62%	±	1.89%
ent-16(*A*)-9-*epi*-ST-Δ^{14}-10-PhytoF	200	67.98%	±	2.87%	84.45%	±	3.40%	57.41%	±	1.92%
ent-16(*B*)-9-*epi*-ST-Δ^{14}-10-PhytoF	200	67.18%	±	3.33%	91.24%	±	4.05%	61.29%	±	2.55%
5(*R*)-5-F_{3t}-IsoP	200	160.07%	±	9.97%	54.38%	±	11.14%	87.05%	±	5.89%
5(*S*)-5-F_{3t}-IsoP	200	87.57%	±	9.44%	41.63%	±	8.66%	36.45%	±	4.00%
8(*R*)-8-F_{3t}-IsoP	200	71.63%	±	4.23%	79.59%	±	3.91%	57.01%	±	1.47%
8(*S*)-8-F_{3t}-IsoP	200	68.77%	±	3.69%	82.92%	±	2.98%	57.03%	±	2.36%
18(*R*)-18-F_{3t}-IsoP	200	72.48%	±	5.71%	72.27%	±	5.40%	52.38%	±	3.17%
4(*RS*)-4-F_{4t}-NeuroP	200	78.15%	±	3.15%	76.29%	±	2.69%	59.62%	±	1.13%

Table S5. The efficiency of sample preparation of Mi134 with the extraction recovery (ER), the matrix effect (ME) and the global process efficiency (PE).

Compound	Concentration (ng/mL)	Extraction Recovery (ER)	±	SD	Matrix Effect (ME)	±	SD	Process Efficiency (PE)	±	SD
D4-10(*R*)-10F_{4t} NeuroP	40	55.54%	±	8.38%	54.81%	±	7.14%	30.44%	±	7.66%
D4-15-F_{2t}-IsoP	40	42.87%	±	6.53%	65.08%	±	4.49%	27.90%	±	7.93%
C21 15F_{2t} IsoP	40	52.93%	±	7.91%	59.89%	±	6.77%	31.70%	±	7.35%
C19 16-F_{1t}-PhytoP	40	37.62%	±	6.82%	62.49%	±	5.51%	23.51%	±	5.89%
5(*RS*)-5-F_{2c}-IsoP	200	42.23%	±	21.23%	66.73%	±	9.58%	28.18%	±	12.26%
5(*RS*)-5-F_{2t}-IsoP	200	49.78%	±	22.63%	60.39%	±	11.91%	30.06%	±	11.72%
15-*epi*-F_{2t}-IsoP	200	37.06%	±	16.36%	59.49%	±	7.82%	22.05%	±	10.04%
15(*RS*)-15-F_{2t}-IsoP	200	41.84%	±	17.09%	69.97%	±	5.98%	29.27%	±	12.45%
2.3-*dinor*-15-F_{2t}-IsoP	200	36.26%	±	18.34%	77.73%	±	6.00%	28.19%	±	14.07%
4-F_{3t}-NeuroP	200	56.41%	±	19.57%	48.37%	±	12.46%	27.29%	±	7.80%
9-F_{1t}-PhytoP	200	29.75%	±	18.83%	81.36%	±	3.39%	24.20%	±	17.04%
ent-16-F_{1t}-PhytoP	200	32.78%	±	21.35%	77.42%	±	8.09%	25.38%	±	15.74%
ent-16-*epi*-16-F_{1t}-PhytoP	200	31.57%	±	18.34%	80.52%	±	4.66%	25.42%	±	15.45%
16B_{1t}-PhytoP	200	44.10%	±	17.67%	70.42%	±	5.92%	31.06%	±	12.27%
9L_{1t}-PhytoP	200	65.16%	±	13.99%	99.10%	±	8.09%	65.16%	±	13.99%
ent-16(*A*)-9-*epi*-ST-Δ^{14}-10-PhytoF	200	32.00%	±	25.01%	72.15%	±	6.30%	23.09%	±	21.15%
ent-16(*B*)-9-*epi*-ST-Δ^{14}-10-PhytoF	200	28.82%	±	24.28%	79.68%	±	3.11%	22.96%	±	24.44%
ent-9(*A*)-12-*epi*-ST-Δ^{10}-13-PhytoF	200	32.41%	±	19.87%	75.76%	±	4.54%	24.55%	±	16.93%
ent-9(*B*)-12-*epi*-ST-Δ^{10}-13-PhytoF	200	31.91%	±	19.57%	70.56%	±	7.81%	22.52%	±	15.97%
5(*S*)-5-F_{3t}-IsoP	200	46.04%	±	22.36%	73.67%	±	9.70%	33.92%	±	15.89%
8(*R*)-8-F_{3t}-IsoP	200	34.49%	±	19.06%	53.79%	±	8.10%	18.55%	±	12.11%
18(*R*)-18-F_{3t}-IsoP	200	39.56%	±	21.55%	76.86%	±	8.09%	30.41%	±	16.32%
4(*RS*)-4-F_{4t}-NeuroP	200	42.01%	±	19.03%	66.63%	±	7.13%	27.99%	±	12.59%
10(*R*)-F_{4t}-NeuroP	200	44.52%	±	18.14%	68.41%	±	7.67%	30.45%	±	11.86%
10(*S*)-F_{4t}-NeuroP	200	39.87%	±	16.43%	64.27%	±	8.74%	25.63%	±	10.67%
13(*A*)-13-F_{4t}-NeuroP	200	50.35%	±	22.67%	64.16%	±	11.79%	32.30%	±	12.96%
13(*B*)-13-F_{4t}-NeuroP	200	38.74%	±	24.05%	67.53%	±	10.88%	26.16%	±	14.03%
14(*R*)-14-F_{4t}-NeuroP	200	44.64%	±	19.04%	61.02%	±	11.20%	27.24%	±	9.98%
14(*S*)-14-F_{4t}-NeuroP	200	50.15%	±	10.07%	61.38%	±	5.65%	30.78%	±	4.94%
20(*R*)-20-F_{4t}-NeuroP	200	50.00%	±	25.04%	61.55%	±	16.56%	30.78%	±	12.80%
20(*S*)-20-F_{4t}-NeuroP	200	45.89%	±	19.81%	64.93%	±	10.39%	29.80%	±	10.13%

Table S6. The efficiency of sample preparation of Mi136 with the extraction recovery (ER), the matrix effect (ME) and the global process efficiency (PE).

Compound	Concentration (ng/mL)	Extraction Recovery (ER)	±	SD	Matrix Effect (ME)	±	SD	Process Efficiency (PE)	±	SD
D4-10(*R*)-10F_{4t} NeuroP	40	82.07%	±	4.21%	51.93%	±	4.91%	42.62%	±	5.72%
D4-15-F_{2t}-IsoP	40	78.51%	±	5.83%	50.73%	±	5.49%	39.83%	±	6.22%
C21 15F_{2t} IsoP	40	90.87%	±	4.54%	55.11%	±	4.91%	50.08%	±	5.83%
C19 16-F_{1t}-PhytoP	40	76.65%	±	4.27%	60.82%	±	3.75%	46.62%	±	5.11%
15-A_{2t}-IsoP	200	68.81%	±	17.28%	89.51%	±	12.58%	61.59%	±	5.72%
5(*RS*)-5-F_{2c}-IsoP	200	81.15%	±	12.65%	37.35%	±	10.39%	30.31%	±	2.87%
5(*RS*)-5-F_{2t}-IsoP	200	98.67%	±	12.21%	43.11%	±	9.65%	42.54%	±	3.57%
15-*epi*-F_{2t}-IsoP	200	82.79%	±	9.39%	33.69%	±	7.53%	27.89%	±	3.36%
15(*RS*)-15-F_{2t}-IsoP	200	79.10%	±	10.33%	39.76%	±	8.37%	31.45%	±	3.30%
2.3-*dinor*-15-F_{2t}-IsoP	200	69.04%	±	11.82%	81.33%	±	11.71%	56.15%	±	7.21%
4-F_{3t}-NeuroP	200	113.11%	±	-58.42%	-0.14%	±	-13.89%	-0.14%	±	13.89%
9-F_{1t}-PhytoP	200	62.59%	±	15.14%	50.80%	±	9.21%	31.79%	±	7.53%
ent-16-*epi*-16-F_{1t}-PhytoP	200	62.09%	±	16.16%	52.12%	±	9.34%	32.36%	±	8.58%
ent-16-F_{1t}-PhytoP	200	60.97%	±	13.78%	37.76%	±	9.00%	23.02%	±	7.26%
16B_{1t}-PhytoP	200	79.07%	±	13.18%	45.73%	±	9.45%	36.16%	±	4.24%
9L_{1t}-PhytoP	200	107.07%	±	11.38%	48.20%	±	8.78%	51.61%	±	3.93%
ent-16(*A*)-13-*epi*-ST-Δ^{14}-9-PhytoF	200	52.89%	±	16.44%	33.31%	±	10.72%	17.62%	±	13.14%
ent-16(*B*)-13-*epi*-ST-Δ^{14}-9-PhytoF	200	55.01%	±	16.85%	43.47%	±	11.73%	23.92%	±	12.55%
ent-16(*A*)-9-*epi*-ST-Δ^{14}-10-PhytoF	200	58.07%	±	17.30%	49.31%	±	9.03%	28.63%	±	10.73%
ent-16(*B*)-9-*epi*-ST-Δ^{14}-10-PhytoF	200	59.93%	±	17.94%	51.42%	±	9.48%	30.81%	±	11.74%
ent-9(*A*)-12-*epi*-ST-Δ^{10}-13-PhytoF	200	52.88%	±	19.14%	48.01%	±	8.80%	25.39%	±	11.93%
ent-9(*B*)-12-*epi*-ST-Δ^{10}-13-PhytoF	200	53.34%	±	19.24%	45.23%	±	11.07%	24.12%	±	12.39%
5(*R*)-5-F_{3t}-IsoP	200	172.34%	±	190.74%	4.75%	±	77.42%	8.19%	±	120.38%
5(*S*)-5-F_{3t}-IsoP	200	58.68%	±	36.37%	58.57%	±	18.26%	34.37%	±	21.34%
8(*R*)-8-F_{3t}-IsoP	200	78.97%	±	12.33%	42.01%	±	9.01%	33.17%	±	4.47%
8(*S*)-8-F_{3t}-IsoP	200	75.60%	±	12.59%	44.72%	±	9.41%	33.81%	±	4.84%
18(*R*)-18-F_{3t}-IsoP	200	74.84%	±	21.46%	47.51%	±	11.23%	35.56%	±	13.08%
18(*S*)-18-F_{3t}-IsoP	200	74.34%	±	14.29%	41.40%	±	10.53%	30.77%	±	5.33%
4(*RS*)-4-F_{4t}-NeuroP	200	85.45%	±	9.81%	37.52%	±	8.25%	32.06%	±	2.24%
10(*R*)-F_{4t}-NeuroP	200	85.66%	±	11.47%	48.81%	±	9.42%	41.81%	±	3.44%
10(*S*)-F_{4t}-NeuroP	200	78.84%	±	12.45%	44.68%	±	10.54%	35.22%	±	4.89%
13(*A*)-13-F_{4t}-NeuroP	200	91.37%	±	11.19%	42.67%	±	9.56%	38.98%	±	3.71%
13(*B*)-13-F_{4t}-NeuroP	200	84.34%	±	13.67%	41.66%	±	10.81%	35.14%	±	3.72%
14(*R*)-14-F_{4t}-NeuroP	200	82.12%	±	10.59%	40.70%	±	9.45%	33.42%	±	3.28%
14(*S*)-14-F_{4t}-NeuroP	200	82.11%	±	9.39%	39.83%	±	8.53%	32.70%	±	1.38%
20(*R*)-20-F_{4t}-NeuroP	200	78.31%	±	11.99%	45.13%	±	10.90%	35.34%	±	5.41%
20(*S*)-20-F_{4t}-NeuroP	200	83.12%	±	11.69%	44.84%	±	9.65%	37.28%	±	2.75%

Table S7. The efficiency of sample preparation of Mi168 with the extraction recovery (ER), the matrix effect (ME) and the global process efficiency (PE).

Compound	Concentration (ng/mL)	Extraction Recovery (ER)	±	SD	Matrix Effect (ME)	±	SD	Process Efficiency (PE)	±	SD
D4-10(*R*)-10F_{4t} NeuroP	40	51.10%	±	6.87%	88.90%	±	5.80%	45.43%	±	7.50%
D4-15-F_{2t}-IsoP	40	43.52%	±	5.21%	78.45%	±	5.04%	34.14%	±	6.06%
C21 15F_{2t} IsoP	40	46.43%	±	5.91%	89.91%	±	5.30%	41.74%	±	6.81%
C19 16-F_{1t}-PhytoP	40	42.30%	±	6.32%	84.63%	±	4.53%	35.80%	±	6.38%
15-A_{2t}-IsoP	200	0.18%	±	46.31%	45.88%	±	6.21%	0.08%	±	41.12%
5(*RS*)-5-F_{2c}-IsoP	200	45.40%	±	6.22%	87.24%	±	2.29%	39.61%	±	4.54%
5(*RS*)-5-F_{2t}-IsoP	200	68.78%	±	6.54%	88.22%	±	3.06%	60.68%	±	4.49%
15-*epi*-F_{2t}-IsoP	200	46.12%	±	4.61%	76.88%	±	1.63%	35.46%	±	4.48%
15(*RS*)-15-F_{2t}-IsoP	200	48.31%	±	4.63%	82.66%	±	2.09%	39.94%	±	3.89%
4-F_{3t}-NeuroP	200	66.12%	±	7.80%	79.63%	±	2.48%	52.65%	±	6.00%
9-F_{1t}-PhytoP	200	36.03%	±	5.34%	85.09%	±	2.98%	30.66%	±	3.97%
ent-16-*epi*-16-F_{1t}-PhytoP	200	35.51%	±	6.38%	89.44%	±	2.84%	31.76%	±	5.31%
ent-16-F_{1t}-PhytoP	200	40.69%	±	5.97%	93.42%	±	3.43%	38.01%	±	5.03%
16B_{1t}-PhytoP	200	65.97%	±	7.17%	86.07%	±	1.59%	56.78%	±	6.10%
9L_{1t}-PhytoP	200	92.23%	±	5.08%	88.92%	±	1.67%	82.02%	±	4.73%
ent-16(*A*)-13-*epi*-ST-Δ^{14}-9-PhytoF	200	61.33%	±	7.74%	84.47%	±	3.23%	51.80%	±	7.00%
ent-16(*B*)-13-*epi*-ST-Δ^{14}-9-PhytoF	200	60.39%	±	7.17%	87.43%	±	2.56%	52.80%	±	8.27%
ent-16(*A*)-9-*epi*-ST-Δ^{14}-10-PhytoF	200	57.74%	±	6.36%	81.58%	±	2.34%	47.10%	±	6.47%
ent-9(*A*)-12-*epi*-ST-Δ^{10}-13-PhytoF	200	59.67%	±	7.66%	82.83%	±	2.32%	49.43%	±	6.94%
ent-9(*B*)-12-*epi*-ST-Δ^{10}-13-PhytoF	200	59.56%	±	7.21%	81.96%	±	2.92%	48.82%	±	8.51%
5(*R*)-5-F_{3t}-IsoP	200	114.06%	±	10.48%	53.15%	±	7.48%	60.63%	±	10.06%
5(*S*)-5-F_{3t}-IsoP	200	55.58%	±	13.08%	79.99%	±	10.81%	44.46%	±	5.50%
8(*R*)-8-F_{3t}-IsoP	200	54.22%	±	6.97%	84.56%	±	2.32%	45.85%	±	5.79%
8(*S*)-8-F_{3t}-IsoP	200	50.15%	±	4.87%	83.39%	±	1.45%	41.82%	±	5.07%
18(*R*)-18-F_{3t}-IsoP	200	49.47%	±	5.76%	88.98%	±	3.53%	44.02%	±	5.08%
18(*S*)-18-F_{3t}-IsoP	200	44.20%	±	6.84%	88.08%	±	2.72%	38.93%	±	5.68%
4(*RS*)-4-F_{4t}-NeuroP	200	63.96%	±	7.57%	85.18%	±	2.05%	54.48%	±	6.20%
10(*R*)-F_{4t}-NeuroP	200	59.77%	±	6.36%	93.16%	±	2.25%	55.68%	±	5.49%
10(*S*)-F_{4t}-NeuroP	200	51.96%	±	5.34%	88.68%	±	3.08%	46.08%	±	5.23%
13(*A*)-13-F_{4t}-NeuroP	200	55.79%	±	5.19%	87.28%	±	2.32%	48.69%	±	4.95%
13(*B*)-13-F_{4t}-NeuroP	200	55.92%	±	7.34%	81.47%	±	1.74%	45.56%	±	6.46%
14(*R*)-14-F_{4t}-NeuroP	200	56.11%	±	6.73%	87.36%	±	3.01%	49.02%	±	5.85%
14(*S*)-14-F_{4t}-NeuroP	200	59.17%	±	6.51%	79.60%	±	2.16%	47.10%	±	4.87%
20(*R*)-20-F_{4t}-NeuroP	200	46.61%	±	4.18%	82.86%	±	2.96%	38.62%	±	5.55%
20(*S*)-20-F_{4t}-NeuroP	200	44.93%	±	4.02%	81.30%	±	1.30%	36.53%	±	3.27%
